# Percutaneous Coronary Intervention in Patients Without Acute Myocardial Infarction in China: Results From the China PEACE Prospective Study of Percutaneous Coronary Intervention

**DOI:** 10.1001/jamanetworkopen.2018.5446

**Published:** 2018-12-14

**Authors:** Yuan Lu, Haibo Zhang, Yongfei Wang, Tianna Zhou, John Welsh, Jiamin Liu, Wenchi Guan, Jing Li, Xi Li, Xin Zheng, John A. Spertus, Frederick A. Masoudi, Harlan M. Krumholz, Lixin Jiang

**Affiliations:** 1 Center for Outcomes Research and Evaluation, Yale University/Yale-New Haven Hospital, New Haven, Connecticut; 2 Section of Cardiovascular Medicine, Department of Internal Medicine, Yale School of Medicine, New Haven, Connecticut; 3 National Clinical Research Center of Cardiovascular Diseases, State Key Laboratory of Cardiovascular Disease, Fuwai Hospital, National Center for Cardiovascular Diseases, Chinese Academy of Medical Sciences and Peking Union Medical College, Beijing, People's Republic of China; 4 NHC Key Laboratory of Clinical Research for Cardiovascular Medications, Beijing, People’s Republic of China; 5 Health Outcomes Research, Saint Luke’s Mid America Heart Institute/University of Missouri-Kansas City, Kansas City; 6 Division of Cardiology, University of Colorado Anschutz Medical Campus, Aurora; 7 Department of Health Policy and Management, Yale School of Public Health, New Haven, Connecticut

## Abstract

**Importance:**

Despite a rapid increase in percutaneous coronary intervention (PCI) procedures in China, little is known about patient-reported health status before and after PCI in patients without acute myocardial infarction (AMI).

**Objective:**

To describe self-perceived angina-specific health status prior to PCI and 1 year after the procedure in patients without AMI in China.

**Design, Setting, and Participants:**

The China Patient-Centered Evaluative Assessment of Cardiac Events (PEACE) Prospective Study of PCI was a population-based, multicenter cohort study of a consecutive sample of 1611 patients without AMI undergoing elective PCI. Participants were enrolled from 40 hospitals in 18 provinces in China from December 2012 to August 2014. Participants were eligible if they underwent PCI for stable and unstable angina and did not have AMI. Participants were excluded if they died in hospital, withdrew from follow-up, or had missing data on self-reported health status at baseline or at 1 year after PCI. The date of the analysis was September 15, 2018.

**Exposures:**

Percutaneous coronary intervention for ischemic heart disease.

**Main Outcomes and Measures:**

Angina frequency and angina-related quality of life were assessed with the Seattle Angina Questionnaire (SAQ) immediately prior to PCI and 1 year after the procedure. Either (1) an increase in the SAQ Angina Frequency score of 10 or more points or (2) an increase in the SAQ Quality-of-Life score of 10 or more points was considered to represent clinically significant improvement.

**Results:**

Of 1611 patients, 520 (32.3%) were women; mean (SD) age was 61.3 (9.8) years. Among these patients, 443 (27.5%) had stable coronary artery disease and 1168 (72.5%) had unstable angina. One hundred fourteen of 443 patients undergoing PCI for stable coronary artery disease (25.7%) and 175 of 1168 undergoing PCI for unstable angina (15.0%) had no reported angina symptoms at the time of the procedure (SAQ Angina Frequency score = 100). Moreover, 18% of all patients (290) had minimal angina symptoms (SAQ Angina Frequency score >90) and, thus, no potential for substantial clinical improvement. Patients with smaller clinical improvements in angina symptom burden at 1 year following PCI had significantly higher baseline SAQ scores for all scales than patients with greater clinical improvement, but generally similar sociodemographic and procedural characteristics.

**Conclusions and Relevance:**

In this study, 25.7% of patients undergoing PCI for stable coronary artery disease had no reported angina symptoms at the time of the procedure. Patients with smaller clinical improvements in angina symptom burden had higher baseline SAQ scores, which highlights the importance of ascertaining impairment from angina among patients without AMI prior to performing PCI.

## Key Points

**Question:**

What is the self-perceived angina-specific health status before and after percutaneous coronary intervention (PCI) among patients without acute myocardial infarction in China?

**Findings:**

In this cohort study of 1611 Chinese patients without acute myocardial infarction undergoing elective PCI, 27.5% had stable coronary artery disease and 72.5% had unstable angina. Of patients undergoing PCI for stable coronary artery disease, 25.7% had no reported angina symptoms at the time of the procedure, and patients with smaller clinical improvements in angina symptom burden at 1 year following PCI had significantly higher baseline Seattle Angina Questionnaire scores for all scales.

**Meaning:**

These findings highlight the importance of ascertaining impairment from angina among patients without acute myocardial infarction prior to performing PCI.

## Teaser

This cohort study uses data from the China Patient-Centered Evaluative Assessment of Cardiac Events (PEACE) Prospective Study to examine the angina-specific health status before and after percutaneous coronary intervention (PCI) in patients without acute myocardial infarction (AMI) in China.

## Introduction

Percutaneous coronary intervention (PCI), one of the most commonly performed cardiac procedures, is primarily indicated to improve symptoms and quality of life when performed for chronic coronary artery disease (CAD).^1,2,3^ Systematically describing patients’ symptom burden before and after PCI can provide important insights on the contemporary selection of patients for the procedure and provide clinicians with the information needed to better set expectations for what patients experience after the procedure. To date, there have been few contemporary descriptions of the symptom burden and quality of life of patients without acute myocardial infarction (AMI) undergoing PCI in routine clinical care globally, and no reports from China, where more than 300 000 procedures were performed in 2011, an 18-fold increase compared with 2001.^4^

Some prior studies have assessed patient selection and PCI appropriateness in high-income countries.^5,6,7,8,9^ These studies have shown that a lack of preprocedural symptoms is associated with an increased rate of inappropriate PCI for nonacute indications. Improvement in patient selection, particularly a decline in the proportions of patients undergoing nonacute PCI who were asymptomatic or had minimal symptoms, has partially led to a reduction in inappropriate PCI in the United States.^5^ However, data are limited from China, where patients may have different demographic and clinical characteristics compared with those in the United States. Furthermore, previous studies have rarely examined the benefits in symptoms and quality of life after the PCI procedure and factors associated with more or less improvement in these patient-centered outcomes. Given the growing emphasis on proper patient selection for coronary procedures to avoid unnecessary procedural risks and improve the value of care, we sought to perform a contemporary analysis of self-perceived health status (angina symptom burden and quality of life) before and after PCI among patients without AMI in China.

In this article, we used data from the China Patient-Centered Evaluative Assessment of Cardiac Events (PEACE) Prospective Study. Our first aim was to describe the characteristics and preprocedural angina-specific health status of patients without AMI who underwent PCI in China. We then assessed these patients’ self-reported health status at 1 year after PCI and the change from baseline.

## Methods

### Participants and Study Design

Details about the design of the China PEACE Prospective Study of PCI have been described previously.^10^ In brief, the study consecutively enrolled 5038 patients undergoing PCI from 40 hospitals in 18 provinces in China from December 2012 to August 2014. Participants were eligible if they received PCI for any indication in a participating hospital. Participants who gave informed consent were then enrolled and followed up at 1, 6, and 12 months. For this analysis, we used data from follow-up at 12 months. We excluded participants with AMI (n = 2317) to focus on those without AMI (ie, stable and unstable angina), for whom relief of angina is a primary goal of PCI. We excluded participants if they did not consent to the study (n = 477) or if they died in hospital or withdrew from follow-up (n = 2). We also excluded participants if they had missing data on self-reported angina frequency or quality of life at baseline (n = 140) or at 1 year after PCI (n = 490). Therefore, a total of 1611 patients were included in the final analysis (eFigure 1 in the Supplement). Approximately 25% of patients were excluded owing to missing data, and most characteristics were similar between patients who were included and those who were excluded (eTable 1 in the Supplement).

The central ethics committee at the China National Center for Cardiovascular Diseases, local internal ethics committees at study sites, and the Yale University institutional review board approved the study. All patients provided written informed consent. The study followed the Strengthening the Reporting of Observational Studies in Epidemiology (STROBE) reporting guideline.

### Clinical Data Collection

At baseline, we collected patients’ demographic and clinical characteristics, as well as self-reported health status (ie, symptoms, functional status, and quality of life) through in-person patient interviews. We obtained patients’ medical histories, treatments, and in-hospital outcomes through central medical record abstraction. We defined unstable angina and stable CAD according to patients’ discharge diagnosis, which relied on physician diagnosis based on patients’ symptom pattern, electrocardiogram results, and clinical status.^11^

We quantified patients’ socioeconomic status from marital status, highest level of education, and health insurance information. We also recorded patients’ cardiovascular risk factors (diabetes mellitus, hypertension, dyslipidemia, current smoker, body mass index, and waist circumference), coexisting conditions (chronic renal dysfunction, acute heart failure, acute stroke, fluid retention, and pneumonia), and medical history (prior MI, coronary artery bypass graft, prior PCI, heart failure, angina pectoris, and stroke). We assessed patients’ baseline clinical presentation by obtaining systolic blood pressure, heart rate, rhythm on electrocardiogram, glomerular filtration rate, Global Registry of Acute Coronary Events risk score,^12^ left ventricular ejection fraction, and number of symptoms at hospital admission. Furthermore, we collected detailed information on the PCI procedure, including arterial access site, number of stents implanted, and the treatment refusal rate. We recorded the medications used during hospitalization and at discharge, as well as patients’ in-hospital outcomes.

At 1 year after discharge from the initial hospital stay, we collected patients’ self-reported health status via either in-person or telephone follow-up interviews. Telephone interviews were conducted when in-person interviews were not feasible (eg, in elderly patients with mobility limitations). Reasons for nonenrollment were documented. Research staff at the China National Center for Cardiovascular Diseases randomly audited 5% of the medical records on an ongoing basis during the enrollment period (between December 2012 and August 2014) and ensured 95% accuracy of the medical record information abstraction.^10^

### Health Status Outcomes

To quantify patients’ angina-specific symptoms and quality of life, we used the Seattle Angina Questionnaire (SAQ), a 19-item, disease-specific questionnaire that has well-established validity and reliability. Scores on the SAQ have proven to be strong predictors of outcomes such as long-term survival, hospitalizations, and costs of care.^13,14,15^ The SAQ quantifies 5 clinically relevant domains of health status, including physical limitation, angina stability, angina frequency, treatment satisfaction, and quality of life. In this study, the SAQ Angina Frequency and Quality-of-Life scales were the primary outcomes. These scales range from 0 to 100, with higher scores indicating fewer symptoms and better quality of life. To facilitate clinical interpretability, ranges of SAQ Angina Frequency scores can be translated qualitatively into daily (0-30), weekly (31-60), monthly (61-99), and no (100) angina. The SAQ Quality-of-Life scores can be translated into very poor to poor (0-24), fair (25-49), good (50-74), and excellent (75-100) quality of life. In our primary analysis, we considered an increase in the SAQ Angina Frequency score of 10 or more points or an increase in the SAQ Quality-of-Life score of 10 or more points as clinically significant improvement. In a sensitivity analysis, to be consistent with prior literature, we considered an increase in the SAQ Angina Frequency score of 20 or more points or an increase in the SAQ Quality-of-Life score of 16 or more points as clinically significant improvement.^16^

### Statistical Analysis

To describe patients’ angina symptom burden prior to PCI, we stratified patients into 4 groups by their diagnosis and self-reported presence of angina: (1) patients with stable CAD and a baseline SAQ Angina Frequency score of 100 (n = 114), (2) patients with unstable angina and a baseline SAQ Angina Frequency score of 100 (n = 175), (3) patients with stable CAD and a baseline SAQ Angina Frequency score of less than 100 (n = 329), and (4) patients with unstable angina and a baseline SAQ Angina Frequency score of less than 100 (n = 993). A baseline SAQ Angina Frequency score of 100 indicated that the patients reported no angina within the prior 4 weeks, whereas a baseline SAQ Angina Frequency score of less than 100 indicated angina symptoms prior to PCI. We compared the demographic and clinical characteristics between these 4 groups. We used χ^2^ tests for categorical variables and Wilcoxon rank sum tests for continuous variables. We also reported the proportion of patients with an SAQ Angina Frequency score greater than 90, as these patients had no potential for clinically significant improvement.

Additionally, within each of these 4 groups, we described the SAQ scores at 1 year following PCI. We described the median with interquartile ranges as well as the percentage of clinically relevant categories for each SAQ score. We also created a scatterplot for patients’ baseline scores against their 1-year scores to visualize the distributions. To describe the 1-year change in patients’ health status, we calculated the differences in baseline and 1-year scores for SAQ Angina Frequency and Quality-of-Life scores. We reported the proportion of patients with clinically significant improvement in Angina Frequency and Quality-of-Life scores.

To evaluate the influence of missing data at baseline, we conducted a sensitivity analysis by creating a best-case scenario in which patients with missing SAQ scores at baseline are assumed to have angina symptoms. To evaluate any potential bias introduced by missing data at 1 year, we conducted a sensitivity analysis using a propensity method. Propensity scores were generated using logistic regression to estimate the probability of missing 1-year SAQ Angina Frequency or Quality-of-Life scores, incorporating baseline demographic and clinical characteristics as predictors.^17^ We then used the inverse of the propensity score to provide 1-year SAQ Angina Frequency or Quality-of-Life scores as a means of weighting the observed responses.^18^ All tests were 2-sided, and *P* < .05 was considered statistically significant. All analyses were done in SAS statistical software version 9.4 (SAS Institute Inc).

## Results

### Baseline Demographic and Clinical Characteristics

A total of 1611 patients from 38 hospitals were included in the analysis. The mean (SD) age was 61.3 (9.8) years and 32.3% (520) were women (Table 1). More than 90% were married and had health insurance either through a public health service, medical insurance for urban workers or residents, or a rural cooperative medical service. These patients had a high prevalence of cardiovascular risk factors, including hypertension (68.5% [1103]), dyslipidemia (50.8% [819]), diabetes (29.1% [469]), and smoking (37.6% [606]). A small proportion of patients had a history of MI (16.1% [260]) or stroke (14.1% [227]), and one-third of patients had a history of heart failure (34.8% [560]).

**Table 1.** Baseline Demographic, Medical History, Clinical Presentation, and Health Status Characteristics by Diagnosis and Baseline SAQ AF Score

Characteristic	Total (N = 1611)	Stable CAD		Unstable Angina		*P* Value
		Baseline SAQ AF = 100 (n = 114)	Baseline SAQ AF <100 (n = 329)	Baseline SAQ AF = 100 (n = 175)	Baseline SAQ AF <100 (n = 993)	
Sociodemographic characteristics, No. (%)						
Age, mean (SD), y	61.3 (9.8)	58.8 (10.6)	62.5 (9.6)	60.1 (8.9)	61.4 (9.8)	.002
Female	520 (32.3)	31 (27.2)	108 (32.8)	51 (29.1)	330 (33.2)	.46
Marriage status						
Married	1489 (92.4)	106 (93)	299 (90.9)	170 (97.1)	914 (92.0)	.41
Divorced or separated	23 (1.4)	3 (2.6)	5 (1.5)	1 (0.6)	14 (1.4)	
Widowed	93 (5.8)	5 (4.4)	23 (7.0)	4 (2.3)	61 (6.1)	
High school education	239 (14.8)	23 (20.2)	40 (12.2)	32 (18.3)	144 (14.5)	.27
Health insurance						
Public health service	56 (3.5)	7 (6.1)	14 (4.3)	4 (2.3)	31 (3.1)	.14
Medical insurance for urban worker or resident	1014 (62.9)	67 (58.8)	202 (61.4)	121 (69.1)	624 (62.8)	
Rural cooperative medical service	461 (28.6)	35 (30.7)	101 (30.7)	39 (22.3)	286 (28.8)	
Other	62 (3.8)	2 (1.8)	9 (2.7)	9 (5.1)	42 (4.2)	
No insurance	16 (1.0)	2 (1.8)	2 (0.6)	2 (1.1)	10 (1.0)	
Cardiovascular risk factors, No. (%)						
Diabetes mellitus	469 (29.1)	45 (39.5)	98 (29.8)	65 (37.1)	261 (26.3)	.002
Hypertension	1103 (68.5)	67 (58.8)	229 (69.6)	123 (70.3)	684 (68.9)	.14
Dyslipidemia	819 (50.8)	64 (56.1)	166 (50.5)	89 (50.9)	500 (50.4)	.71
Current smoker	606 (37.6)	42 (36.8)	116 (35.3)	84 (48.0)	364 (36.7)	.03
Body mass index, median (IQR)^a^	25 (23.0-27.1)	25 (23.4-26.9)	25 (22.9-27.1)	26 (23.4-27.9)	25 (22.9-27.1)	.02
Body mass index^a^						
≤28	1116 (69.3)	79 (69.3)	230 (69.9)	109 (62.3)	698 (70.3)	.16
>28	241 (15.0)	13 (11.4)	55 (16.7)	35 (20.0)	138 (13.9)	
Waist circumference, median (IQR), cm	90 (83.0-95.5)	90 (85.0-97.0)	90 (83.0-95.0)	90 (83.0-97.0)	90 (82.5-95.5)	.14
Coexisting conditions, No. (%)						
Acute heart failure	15 (0.9)	0	6 (1.8)	0	9 (0.9)	.14
Acute stroke	35 (2.2)	3 (2.6)	8 (2.4)	4 (2.3)	20 (2.0)	.95
Fluid retention (lower extremity edema)	84 (5.2)	5 (4.4)	24 (7.3)	4 (2.3)	51 (5.1)	.11
Pneumonia	53 (3.3)	3 (2.6)	13 (4.0)	3 (1.7)	34 (3.4)	.57
Medical history, No. (%)						
Prior myocardial infarction	260 (16.1)	32 (28.1)	68 (20.7)	29 (16.6)	131 (13.2)	<.001
Prior coronary artery bypass graft	12 (0.7)	0	3 (0.9)	1 (0.6)	8 (0.8)	.78
Prior PCI	264 (16.4)	27 (23.7)	62 (18.8)	30 (17.1)	145 (14.6)	.04
Prior heart failure	560 (34.8)	35 (30.7)	113 (34.3)	47 (26.9)	365 (36.8)	.06
Prior angina pectoris	218 (13.5)	10 (8.8)	41 (12.5)	18 (10.3)	149 (15)	.11
Prior stroke	227 (14.1)	12 (10.5)	42 (12.8)	29 (16.6)	144 (14.5)	.44
Vital and laboratory results						
Systolic blood pressure, median (IQR), mm Hg	130 (120.0-146.0)	130 (120.0-140.0)	130 (120.0-145.0)	140 (125.0-150.0)	130 (120.0-145.0)	.15
Heart rate on admission, median (IQR), beats/min	70 (64.0-78.0)	71 (65.0-79.0)	74 (66.0-80.0)	70 (64.0-78.0)	70 (63.0-78.0)	.02
Electrocardiogram findings, No. (%)						
Rhythm on electrocardiogram						
Atrial fibrillation or flutter	37 (2.3)	4 (3.5)	9 (2.7)	2 (1.1)	22 (2.2)	.55
Ventricular tachycardia	3 (0.2)	0	1 (0.3)	0	2 (0.2)	.85
Left bundle branch block	10 (0.6)	0	0	0	10 (1.0)	.10
Left ventricular ejection fraction, median (IQR), %	62 (57-67)	60 (57-66)	62 (58-67)	61 (55-65)	62 (58-66)	.21
Glomerular filtration rate, median (IQR), mL/min/1.73 m^2^	79 (69.3-91.2)	80 (66.8-94.5)	80 (69.9-89.5)	81 (67.8-94.4)	79 (69.3-90.9)	.50
Disease severity						
Global Registry of Acute Coronary Events risk score, median (IQR)	103 (85.0-121.0)	99 (83.0-122.0)	106 (88.0-124.0)	96 (82.0-116.0)	103 (85.0-120.0)	.05
Symptoms, median (IQR), No.	2 (1-4)	1 (1-4)	2 (1-4)	2 (1-3)	2 (1-3)	<.001
Health status on admission, No. (%)						
SAQ Physical Limitation score, median (IQR)	83 (66.7-100.0)	100 (86.1-100.0)	83 (63.9-100.0)	92 (72.2-100.0)	83 (65.3-95.8)	<.001
SAQ Physical Limitation, No. (%)						
Minimal (>75)	950 (59.0)	91 (79.8)	188 (57.1)	122 (69.7)	549 (55.3)	<.001
Mild (>50 and ≤75)	412 (25.6)	14 (12.3)	77 (23.4)	42 (24.0)	279 (28.1)	
Moderate (>25 and ≤50)	151 (9.4)	4 (3.5)	42 (12.8)	4 (2.3)	101 (10.2)	
Severe (≤25)	98 (6.1)	5 (4.4)	22 (6.7)	7 (4.0)	64 (6.4)	
SAQ Angina Stability score, median (IQR)	25 (0-50.0)	50 (50.0-50.0)	25 (0-50.0)	50 (50.0-50.0)	25 (0-50.0)	<.001
SAQ Angina Stability, No. (%)						
Much better (>75)	149 (9.2)	4 (3.5)	40 (12.2)	17 (9.7)	88 (8.9)	<.001
Slightly better (>50 and ≤75)	46 (2.9)	1 (0.9)	18 (5.5)	3 (1.7)	24 (2.4)	
Unchanged (50)	505 (31.3)	98 (86.0)	76 (23.1)	139 (79.4)	192 (19.3)	
Slightly worse (≥25 and <50)	408 (25.3)	3 (2.6)	85 (25.8)	12 (6.9)	308 (31.0)	
Much worse (<25)	503 (31.2)	8 (7.0)	110 (33.4)	4 (2.3)	381 (38.4)	
SAQ Angina Frequency score, median (IQR)	60 (40-80)	100 (100-100)	50 (20-70)	100 (100-100)	60 (20-70)	<.001
SAQ Angina Frequency, No. (%)						
None (100)	289 (17.9)	114 (100)	0	175 (100)	0	<.001
Monthly (>60 and <100)	418 (25.9)	0	96 (29.2)	0	322 (32.4)	
Weekly (>30 and ≤60)	514 (31.9)	0	127 (38.6)	0	387 (39.0)	
Daily (≤30)	390 (24.2)	0	106 (32.2)	0	284 (28.6)	
SAQ Quality-of-Life score, median (IQR)	58 (41.7-75.0)	67 (58.3-91.7)	58 (41.7-75.0)	75 (50.0-83.3)	50 (33.3-66.7)	<.001
SAQ Quality of Life, No. (%)						
Excellent (>75)	447 (27.7)	54 (47.4)	96 (29.2)	89 (50.9)	208 (20.9)	<.001
Good (>50 and ≤75)	649 (40.3)	46 (40.4)	133 (40.4)	49 (28.0)	421 (42.4)	
Fair (>25 and ≤50)	390 (24.2)	12 (10.5)	81 (24.6)	32 (18.3)	265 (26.7)	
Poor to very poor (≤25)	125 (7.8)	2 (1.8)	19 (5.8)	5 (2.9)	99 (10.0)	

Abbreviations: AF, angina frequency; CAD, coronary artery disease; IQR, interquartile range; PCI, percutaneous coronary intervention; SAQ, Seattle Angina Questionnaire.

^a^ Calculated as weight in kilograms divided by height in meters squared.

Regarding the PCI procedure, the most common access site was through the radial artery (89.8% [1446]), followed by the femoral artery (7.8% [125]) and other sites (1.4% [9]) (Table 2). The mean (SD) number of stents implanted was 1.76 (0.95). More than 90% of patients received aspirin and more than 95% received statins, clopidogrel, or heparin during their hospitalizations. A majority of patients were administered antianginal medications such as β-blockers (73.3% [1181]) and nitrates (90.2% [1453]) during hospitalization and a majority of patients were discharged with β-blockers (60.6% [976]). Nitrates, calcium channel blockers, glycoprotein IIb/IIIa inhibitors, low-molecular-weight heparin, and fondaparinux were less commonly prescribed during hospitalizations and were received by 29.2% (471), 28.8% (464), 20.9% (337), 29.1% (468), and 17.6% (283) of patients, respectively. In-hospital complication rates were low: 3.5% of patients (57) had bleeding of some degree while hospitalized, 1.4% (22) experienced recurrent MI, 1.2% (19) experienced atrial fibrillation, and 3.6% (58) experienced an ischemic stroke (Table 3). The mean (SD) length of hospital stay was 11.2 (5.2) days.

**Table 2.** Percutaneous Coronary Intervention Procedure and Treatment Information by Diagnosis and Baseline SAQ AF Score^a^

Characteristic	No. (%)					*P* Value
	Total (N = 1611)	Stable CAD		Unstable Angina		
		Baseline SAQ AF = 100 (n = 114)	Baseline SAQ AF <100 (n = 329)	Baseline SAQ AF = 100 (n = 175)	Baseline SAQ AF <100 (n = 993)	
Arterial access site						
Femoral artery	125 (7.8)	10 (8.8)	26 (7.9)	15 (8.6)	74 (7.5)	.76
Radial artery	1446 (89.8)	103 (90.4)	295 (89.7)	153 (87.4)	895 (90.1)	
Brachial artery	14 (0.9)	0	4 (1.2)	3 (1.7)	7 (0.7)	
Other	9 (0.6)	0	0	1 (0.6)	8 (0.8)	
Unrecorded	17 (1.1)	1 (0.9)	4 (1.2)	3 (1.7)	9 (0.9)	
Stents implanted, mean (SD), No.	1.8 (1.0)	1.7 (0.8)	1.8 (1.0)	1.8 (0.9)	1.8 (1.0)	.65
Recommended and refused treatments						
Recommended PCI	1517 (94.2)	107 (93.9)	321 (97.6)	163 (93.1)	926 (93.3)	.03
Recommended coronary artery bypass graft	58 (3.6)	2 (1.8)	3 (0.9)	7 (4.0)	46 (4.6)	.01
Refused coronary artery bypass graft	64 (4.0)	4 (3.5)	6 (1.8)	9 (5.1)	45 (4.5)	.14
In-hospital medication						
Aspirin	1476 (91.6)	103 (90.4)	313 (95.1)	155 (88.6)	905 (91.1)	.05
β-Blocker	1181 (73.3)	83 (72.8)	243 (73.9)	124 (70.9)	731 (73.6)	.74
Statin	1574 (97.7)	109 (95.6)	319 (97.0)	173 (98.9)	973 (98.0)	.24
Clopidogrel	1598 (99.2)	114 (100)	326 (99.1)	175 (100)	983 (99.0)	.46
Glycoprotein IIb/IIIa inhibitor	337 (20.9)	27 (23.7)	57 (17.3)	46 (26.3)	207 (20.8)	.21
Heparin	1534 (95.2)	104 (91.2)	316 (96.0)	166 (94.9)	948 (95.5)	.13
Low-molecular-weight heparin	468 (29.1)	24 (21.1)	93 (28.3)	43 (24.6)	308 (31.0)	.12
Fondaparinux	283 (17.6)	20 (17.5)	52 (15.8)	33 (18.9)	178 (17.9)	.74
Nitrates	1453 (90.2)	91 (79.8)	281 (85.4)	159 (90.9)	922 (92.8)	<.001
Calcium channel blocker	464 (28.8)	31 (27.2)	103 (31.3)	46 (26.3)	284 (28.6)	.63
Discharge medication						
Aspirin	1196 (74.2)	98 (86.0)	274 (83.3)	118 (67.4)	706 (71.1)	<.001
β-Blocker	976 (60.6)	77 (67.5)	227 (69.0)	102 (58.3)	570 (57.4)	.002
Statin	1238 (76.8)	95 (83.3)	276 (83.9)	126 (72.0)	741 (74.6)	.005
Nitrates	471 (29.2)	27 (23.7)	85 (25.8)	42 (24.0)	317 (31.9)	<.001

Abbreviations: AF, angina frequency; CAD, coronary artery disease; PCI, percutaneous coronary intervention; SAQ, Seattle Angina Questionnaire.

**Table 3.** In-Hospital Outcomes by Diagnosis and Baseline SAQ AF Score

Outcome	No (%)					*P* Value
	Total (N = 1611)	Stable CAD		Unstable Angina		
		Baseline SAQ AF = 100 (n = 114)	Baseline SAQ AF <100 (n = 329)	Baseline SAQ AF = 100 (n = 175)	Baseline SAQ AF <100 (n = 993)	
Cardiac-related complication						
Recurrent MI^a^	22 (1.4)	2 (1.8)	8 (2.4)	2 (1.1)	10 (1.0)	.27
Chronic heart failure exacerbation	1 (0.1)	0	0	0	1 (0.1)	.89
Cardiopulmonary resuscitation	1 (0.1)	0	1 (0.3)	0	0	.27
Atrial flutter or atrial fibrillation	19 (1.2)	3 (2.6)	6 (1.8)	0	10 (1.0)	.13
Ventricular fibrillation	2 (0.1)	0	1 (0.3)	0	1 (0.1)	.74
Bleeding						
Major bleeding	0	0	0	0	0	NA
Minor bleeding	20 (1.2)	2 (1.8)	3 (0.9)	4 (2.3)	11 (1.1)	.52
Other bleeding	37 (2.3)	1 (0.9)	13 (4.0)	1 (0.6)	22 (2.2)	.06
Ischemic stroke	58 (3.6)	5 (4.4)	14 (4.3)	9 (5.1)	30 (3)	.43
Acute renal failure	2 (0.1)	0	0	0	2 (0.2)	.74
Dialysis	2 (0.1)	0	0	1 (0.6)	1 (0.1)	.33
Deep venous thrombosis	1 (0.1)	0	0	0	1 (0.1)	.89
Dissection	6 (0.4)	0	3 (0.9)	1 (0.6)	2 (0.2)	.26
Hematoma	20 (1.2)	0	5 (1.5)	2 (1.1)	13 (1.3)	.64
Length of stay, mean (SD), d	11.2 (5.2)	11.6 (6.2)	11.3 (4.8)	11.5 (7.1)	11.1 (4.8)	.48

Abbreviations: AF, angina frequency; CAD, coronary artery disease; MI, myocardial infarction; NA, not applicable; SAQ, Seattle Angina Questionnaire.

^a^ Defined as acute MI that occurred during hospitalization among patients with a history of MI.

### Angina Symptoms Prior to PCI

Among all patients, 27.5% (443) had stable CAD and 72.5% (1168) had unstable angina. One hundred fourteen of 443 patients with stable CAD (25.7%) and 175 of 1168 patients with unstable angina (15.0%) had no reported angina symptoms within 4 weeks prior to the procedure (SAQ Angina Frequency score = 100). Three hundred twenty-nine patients with stable CAD (74.3%) and 993 of those with unstable angina (85.0%) had reported some degree of chronic symptoms (SAQ Angina Frequency score <100). For all patients, 18% (290) had minimal symptoms (SAQ Angina Frequency score, 90-100) and, thus, no potential for substantial clinical improvement (Table 1, Table 2, and Table 3). In a sensitivity analysis in which we used 20 points as the threshold for clinical improvement, 24.8% of patients had an SAQ Angina Frequency score greater than 80 and, thus, no potential for improvement.

Compared with patients who had no reported angina symptoms within the prior 4 weeks, patients who had reported some degree of chronic symptoms were more likely to be older, be female, and have prior angina, higher Global Registry of Acute Coronary Events scores, more symptoms, more physical limitations, and lower quality of life.

A total of 4.6% of patients had missing data on baseline angina symptoms prior to PCI. In a sensitivity analysis in which we assumed all such patients had clinically significant symptoms, 24.6% of people undergoing PCI for stable CAD and 14.3% of people undergoing PCI for unstable angina would have had no symptoms within 4 weeks prior to the procedure.

### Angina Symptoms and Quality of Life 1 Year After PCI

At 1 year after PCI, considerable heterogeneity existed in the intraindividual changes in health status (Figure and eFigure 2 in the Supplement). Among patients with stable CAD who reported no angina symptoms at baseline, 14% (16) had reported some degree of angina symptom burden at 1 year (Table 4). The proportions of patients who reported excellent, good, fair, and poor to very poor quality of life were 48.2% (55), 39.5% (45), 10.5% (12), and 1.8% (2), respectively. A third of patients (32.5% [37]) had clinically improved quality of life (change in SAQ Quality-of-Life score ≥10 points) at 1 year, while 67.5% (77) of the patients did not experience clinically improved quality of life. More than 90% of patients reported unchanged angina stability and minimal physical limitation.

**Figure.**
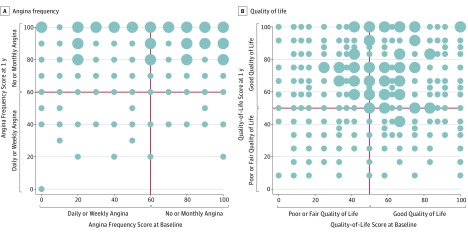
Angina Frequency and Quality of Life Scatterplots of Seattle Angina Questionnaire Angina Frequency score (A) and Quality-of-Life score (B) at baseline hospitalization and at 1 year after percutaneous coronary intervention. The size of the dot indicates the number of patients, with smaller dots indicating fewer than 16 patients and larger dots indicating 16 or more patients.

**Table 4.** Seattle Angina Questionnaire Health Status 1 Year After PCI by Diagnosis and Baseline SAQ AF Score

Health Status	Total (N = 1611)	Stable CAD		Unstable Angina		*P* Value
		Baseline SAQ AF = 100 (n = 114)	Baseline SAQ AF <100 (n = 329)	Baseline SAQ AF = 100 (n = 175)	Baseline SAQ AF <100 (n = 993)	
SAQ Physical Limitation score, median (IQR)	100 (80.6-100.0)	100 (86.1-100.0)	94.4 (75.0-100.0)	100 (75.0-100.0)	100 (83.3-100.0)	.04
SAQ Physical Limitation, No. (%)						
Minimal (>75)	1227 (76.2)	91 (79.8)	234 (71.1)	128 (73.1)	774 (77.9)	.14
Mild (>50 and ≤75)	278 (17.3)	19 (16.7)	73 (22.2)	35 (20.0)	151 (15.2)	
Moderate (>25 and ≤50)	44 (2.7)	1 (0.9)	8 (2.4)	7 (4.0)	28 (2.8)	
Severe (≤25)	62 (3.8)	3 (2.6)	14 (4.3)	5 (2.9)	40 (4.0)	
SAQ Angina Stability score, median (IQR)	50 (50-50)	50 (50-50)	50 (50-50)	50 (50-50)	50 (50-50)	.06
SAQ Angina Stability, No. (%)						
Much better (>75)	290 (18.0)	12 (10.5)	60 (18.2)	26 (14.9)	192 (19.3)	.19
Slightly better (>50 and ≤75)	55 (3.4)	2 (1.8)	14 (4.3)	3 (1.7)	36 (3.6)	
Unchanged (50)	1202 (74.6)	98 (86.0)	238 (72.3)	138 (78.9)	728 (73.3)	
Slightly worse (≥25 and <50)	49 (3.0)	1 (0.9)	14 (4.3)	7 (4.0)	27 (2.7)	
Much worse (<25)	15 (0.9)	1 (0.9)	3 (0.9)	1 (0.6)	10 (1.0)	
SAQ Angina Frequency score, median (IQR)	100 (90-100)	100 (100-100)	100 (90-100)	100 (100-100)	100 (90-100)	<.001
SAQ Angina Frequency, No. (%)						
None (100)	1167 (72.4)	98 (86)	223 (67.8)	135 (77.1)	711 (71.6)	.01
Monthly (>60 and <100)	344 (21.4)	12 (10.5)	76 (23.1)	31 (17.7)	225 (22.7)	
Weekly (>30 and ≤60)	88 (5.5)	3 (2.6)	25 (7.6)	9 (5.1)	51 (5.1)	
Daily (≤30)	12 (0.7)	1 (0.9)	5 (1.5)	0	6 (0.6)	
SAQ Quality-of-Life score, median (IQR)	66.7 (50.0-87.5)	66.7 (58.3-91.7)	75 (58.3-91.7)	75 (58.3-100.0)	66.7 (50.0-83.3)	<.001
SAQ Quality of Life, No. (%)						
Excellent (>75)	804 (49.9)	55 (48.2)	174 (52.9)	109 (62.3)	466 (46.9)	.01
Good (>50 and ≤75)	551 (34.2)	45 (39.5)	117 (35.6)	44 (25.1)	345 (34.7)	
Fair (>25 and ≤50)	233 (14.5)	12 (10.5)	34 (10.3)	20 (11.4)	167 (16.8)	
Poor to very poor (≤25)	23 (1.4)	2 (1.8)	4 (1.2)	2 (1.1)	15 (1.5)	

Abbreviations: AF, angina frequency; CAD, coronary artery disease; IQR, interquartile range; SAQ, Seattle Angina Questionnaire.

Among patients with unstable angina who reported no angina symptoms at baseline, 23% (40) reported some degree of angina symptom burden at 1 year. The proportions of patients who reported excellent, good, fair, and poor to very poor quality of life were 62.3% (109), 25.1% (44), 11.4% (20), and 1.1% (2), respectively. Of these patients, 38.9% (68) had clinically improved quality of life (change in SAQ Quality-of-Life score ≥10 points) at 1 year, while 61.1% (107) did not experience clinically improved quality of life. Slightly less than 80% of patients reported unchanged angina stability and minimal physical limitation.

Among patients with stable CAD who reported some degree of angina symptoms at baseline, 67.8% (223) had no reported angina symptom burden, 23.1% (76) had monthly symptoms, and 8.1% (30) had weekly or daily symptoms at 1 year following PCI. More than 90% of patients (90.9% [299]) had clinically improved angina frequency (change in SAQ Angina Frequency score ≥10 points) at 1 year, while 9.1% of the patients (30) did not experience clinically improved angina frequency. The proportions of patients who reported excellent, good, fair, and poor to very poor quality of life were 52.9% (174), 35.6% (117), 10.3% (34), and 1.2% (4), respectively. About half of patients (53.2% [175]) had clinically improved quality of life (change in SAQ Quality-of-Life score ≥10 points) at 1 year, while 46.8% of patients (154) did not experience clinically improved quality of life. Of patients with stable CAD who reported some degree of angina symptoms at baseline, 72.3% (238) reported unchanged angina stability, followed by 22.5% (74) with better angina stability and 5.2% (17) with worse angina stability. More than 90% of patients reported minimal or mild physical limitation.

Among patients with unstable angina who reported some degree of angina symptoms at baseline, 71.6% (771) had no reported angina symptom burden, 22.7% (225) had monthly symptoms, and 5.7% (57) had weekly or daily symptoms 1 year after PCI. More than 90% of patients (91.8% [912]) had clinically improved angina frequency (change in SAQ Angina Frequency score ≥10 points) at 1 year, while 8.2% of the patients (81) did not experience clinically improved angina frequency. The proportions of patients who reported excellent, good, fair, and poor to very poor quality of life were 46.9% (446), 34.7% (345), 16.8% (167), and 1.5% (15), respectively. About half of patients (54% [536]) had clinically improved quality of life (change in SAQ Quality-of-Life score ≥10 points) at 1 year, while 46% of patients (457) did not experience clinically improved quality of life. Of patients with unstable angina who reported some degree of angina symptoms at baseline, 73.8% (728) reported unchanged angina stability, followed by 22.9% (228) with better angina stability and 3.7% (37) with worse angina stability. More than 90% of patients reported minimal or mild physical limitation.

Compared with patients who had higher clinical improvements in angina symptoms, those with smaller clinical improvements had significantly higher baseline SAQ scores for all scales (eTable 2 in the Supplement). The sensitivity analysis accounting for missing data in SAQ scores at 1 year showed similar results as the observed data, suggesting a lack of bias in the observed data (eTable 3 in the Supplement).

## Discussion

In this study, 25.7% of patients with stable CAD undergoing PCI in China reported no angina symptoms at the time of the procedure. Moreover, many patients had minimal angina symptoms. In our secondary analyses we showed that patients with smaller clinical improvements had significantly higher baseline SAQ scores for all scales. While this may be expected, it also highlights the importance of ascertaining impairment from angina prior to performing PCI among patients without AMI, given that the primary goal of elective PCI is to improve patients’ symptoms.

Our study extends the prior literature in several important ways. Previous studies have primarily focused on clinical outcomes such as death and adverse events and did not collect data on patient-reported outcomes over time.^19,20,21,22,23,24,25,26,27,28,29^ Our study extends the work of Arnold et al,^30^ who explicitly described the proportions of patients who derived both an angina and a quality-of-life benefit from treatment, thereby demonstrating that baseline SAQ Angina Frequency and Quality-of-Life scores were the strongest predictors of being angina free after PCI. We also found that a substantial proportion of patients have minimal or no angina symptoms prior to PCI and, thus, no potential for substantial clinical improvement. Although future research is needed to better quantify the heterogeneity in the benefits of PCI, these results have implications not only for informing patients without AMI about PCI’s benefits or lack thereof, in light of patients’ misconceptions and overestimates of any potential benefits of PCI, but also for identifying opportunities to improve patient outcomes.^31,32^

Our findings are clinically relevant and can help clinicians and patients understand which patients are most likely to benefit (or not) from PCI, thereby strengthening clinicians’ ability to convey the low likelihood of benefit to minimally symptomatic patients in the context of shared decision making. These findings also suggest that the assessment of preprocedural health status scores is critical for patient selection and for setting realistic expectations for patients. In addition, our study evaluated a substantial range of patient characteristics (eg, demographic, clinical, behavioral, medication, and hospital care) and hospital attributes that have not been collected in previous studies. This allowed us to better characterize patients without AMI who derived fewer quality-of-life benefits from PCI. Our finding will inform future multivariable analyses of baseline and clinical characteristics independently associated with low benefits from PCI. Such information is particularly important in developing countries such as China, where there are relatively fewer medical resources and not enough interventional cardiologists to perform these procedures. Identifying patients who are not likely to benefit from PCI averts waste in the health system and the potential risks associated with the procedure.

### Limitations

Our findings should be interpreted in the context of several potential limitations. First, our study may not be nationally representative. However, our enrollment of patients from 40 hospitals in 18 provinces encompasses disparate regions of China. Second, although only 75% of the patients enrolled at baseline completed the 1-year follow-up, our rates of missing data were in line with other high-quality studies such as TRIUMPH (Translational Research Investigating Underlying Disparities in Acute Myocardial Infarction Patients’ Health Status)^33^ and PREMIER (Prospective Registry Evaluating Myocardial Infarction: Events and Recovery).^34^ Our sensitivity analyses using an inverse probability weighting approach showed that missing data introduced no significant bias to the results, which suggests that the observed data were representative of the entire population that we studied. Third, our study focused on angina-specific changes in symptom burden and did not examine other nonanginal symptoms. For patients with a diagnosis of unstable angina who reported no angina over the 4 weeks prior to PCI, 93% (163) had nonanginal symptoms, such as dyspnea, nausea, fatigue, and pain radiation to the back, shoulder, arm, and finger. The presence of nonanginal symptoms may lead to the diagnosis and management of unstable angina. It is also possible that physicians misclassified patients’ diagnosis as unstable angina, which could not be independently adjudicated.

## Conclusions

Our findings identified 25.7% of patients undergoing PCI for stable CAD who had no reported angina symptoms at the time of the procedure. Moreover, many patients had minimal angina symptoms. Patients with smaller clinical improvements in angina symptom burden at 1 year following PCI had significantly higher baseline SAQ scores for all scales, highlighting the importance of ascertaining impairment from angina among patients without AMI prior to performing PCI.

## Notes

Supplement. **eTable 1. **Comparison of Patients Included in Analysis and Patients Excluded Due to Missing Data on SAQ Angina Frequency and Quality-of-Life at 1 Year **eTable 2.** Baseline Demographic, Medical History, Clinical Presentation, and Health Status Characteristics, Overall and Compared Between Patients With and Without Clinically Significant Improvement of SAQ Angina Frequency and Quality-of-Life Over 1 Year **eTable 3.** SAQ Score With and Without Adjusting for Missing Data **eFigure 1.** Flowchart of Study Population Selection **eFigure 2.** Density Plot of 1-Year Change in SAQ Angina Frequency and Quality-of-Life Scores
