# Supplementary material for: Percutaneous Coronary Intervention in Patients Without Acute Myocardial Infarction in China: Results From the China PEACE Prospective Study of Percutaneous Coronary Intervention
Source: JAMA Netw Open. 2018 Dec 14;1(8):e185446. doi: 10.1001/jamanetworkopen.2018.5446 (PMC6324328; doi:10.1001/jamanetworkopen.2018.5446)
Supplement: Supplement. — eTable 1. Comparison of Patients Included in Analysis and Patients Excluded Due to Missing Data on SAQ Angina Frequency and Quality-of-Life at 1 Year eTable 2. Baseline Demographic, Medical History, Clinical Presentation, and Health Status Characteristics, Overall and Compared Between Patients With and Without Clinically Significant Improvement of SAQ Angina Frequency and Quality-of-Life Over 1 Year eTable 3. SAQ Score With and Without Adjusting for Missing Data eFigure 1. Flowchart of Study Population Selection eFigure 2. Density Plot of 1-Year Change in SAQ Angina Frequency and Quality-of-Life Scores [file jamanetwopen-1-e185446-s001.pdf]

Supplementary Online Content

Lu Y, Zhang H, Wang Y, et al. Percutaneous coronary intervention in patients without acute myocardial infarction in China: results from the China PEACE Prospective Study of Percutaneous Coronary Intervention. *JAMA Netw Open*. 2018;1(8):e185446. doi:10.1001/jamanetworkopen.2018.5446

eTable 1. Comparison of Patients Included in Analysis and Patients Excluded Due to Missing Data on SAQ Angina Frequency and Quality-of-Life at 1 Year

eTable 2. Baseline Demographic, Medical History, Clinical Presentation, and Health Status Characteristics, Overall and Compared Between Patients With and Without Clinically Significant Improvement of SAQ Angina Frequency and Quality-of-Life Over 1 Year

eTable 3. SAQ Score With and Without Adjusting for Missing Data

eFigure 1. Flowchart of Study Population Selection

eFigure 2. Density Plot of 1-Year Change in SAQ Angina Frequency and Quality-of-Life Scores

This supplementary material has been provided by the authors to give readers additional information about their work.

© 2018 Lu Y et al. *JAMA Network Open.*

eTable 1. Comparison of patients included in analysis and patients excluded due to missing data on SAQ Angina Frequency and Quality-of-Life at 1 year[a]

Characteristics	Total (N = 2242)	Patients included in the analysis (n = 1611)	Patients excluded due to missing data on angina frequency or quality of life (n = 631)	P value
**Sociodemographics characteristics, No. (%)**				
Age, y: Mean (SD)	62.3 (10.0)	61.3 (9.8)	64.8 (10.2)	<.001
Female	749 (33.4)	520 (32.3)	229 (36.3)	0.07
Marriage Status				
Married	2031 (90.6)	1489 (92.4)	542 (85.9)	<.001
Divorced or separated	31 (1.4)	23 (1.4)	8 (1.3)	
Widowed	160 (7.1)	93 (5.8)	67 (10.6)	
High school education	313 (14.0)	239 (14.8)	74 (11.7)	<.001
Health insurance				
Public health service	70 (3.1)	56 (3.5)	14 (2.2)	0.15
Medical insurance for urban worker or resident	1391 (62.0)	1014 (62.9)	377 (59.8)	
Rural cooperative medical service	672 (30.0)	461 (28.6)	211 (33.4)	
Other	87 (3.9)	62 (3.9)	25 (4.0)	
No insurance	19 (0.9)	16 (1.0)	3 (0.5)	
**Cardiovascular risk factors, No. (%)**				
Diabetes mellitus	674 (30.1)	469 (29.1)	205 (32.5)	0.12
Hypertension	1548 (69.1)	1103 (68.5)	445 (70.5)	0.34
Dyslipidemia	1116 (49.8)	819 (50.8)	297 (47.1)	0.11
Current smoker	830 (37.0)	606 (37.6)	224 (35.5)	0.35
Body mass index, median[a] (IQR)	25 (22.7-27.1)	25 (23.0-27.1)	25 (22.0-27.0)	0.04
Body mass index [a]				
≤28	1527 (68.1)	1116 (69.3)	411 (65.1)	<.001
>28	316 (14.1)	241 (15.0)	75 (11.9)	
Waist circumference, median (IQR), cm	90 (82.0-95.5)	90 (83.0-95.5)	88 (80.0-95.3)	0.03
**Coexisting Conditions, No. (%)**				
Acute heart failure	18 (0.8)	15 (0.9)	3 (0.5)	0.28
Acute Stroke	53 (2.4)	35 (2.2)	18 (2.9)	0.34
Fluid retention (lower extremity edema)	135 (6.0)	84 (5.2)	51 (8.1)	0.01
Pneumonia	84 (3.8)	53 (3.3)	31 (4.9)	0.07
**Medical history, No. (%)**				
Prior myocardial infarction	375 (16.7)	260 (16.1)	115 (18.2)	0.23
Prior coronary artery bypass graft	15 (0.7)	12 (0.7)	3 (0.5)	0.48
Prior PCI	373 (16.6)	264 (16.4)	109 (17.3)	0.61
Prior heart failure	773 (34.5)	560 (34.8)	213 (33.8)	0.65
Prior angina pectoris	283 (12.6)	218 (13.5)	65 (10.3)	0.04
Prior stroke	356 (15.9)	227 (14.1)	129 (20.4)	<.001

Abbreviations: IQR, interquartile range; PCI, percutaneous coronary intervention

© 2018 Lu Y et al. *JAMA Network Open.*

a: Calculated as weight in kilograms divided by height in meters squared.

© 2018 Lu Y et al. *JAMA Network Open.*

eTable 2. Baseline demographic, medical history, clinical presentation, and health status characteristics, overall and compared between patients with and without clinically significant improvement of SAQ Angina Frequency and Quality-of-Life over 1 year[a]

Characteristics	Total (N = 1611)	SAQ Angina Frequency: No significant improvement (n = 400)	SAQ Angina Frequency: Significant improvement (n = 1211)	P value	SAQ Quality of Life: No significant improvement (n = 795)	SAQ Quality of Life: Significant improvement (n = 816)	P value
**Sociodemographic characteristics, No. (%)**							
Age, y: Mean (SD)	61.3 (9.8)	60.6 (9.8)	61.5 (9.8)	0.10	61.2 (9.8)	61.4 (9.8)	0.64
Female	520 (32.3)	126 (31.5)	394 (32.5)	0.70	252 (31.7)	268 (32.8)	0.62
Marriage Status				0.22			0.04
Married	1489 (92.4)	378 (94.5)	1111 (91.7)		724 (91.1)	765 (93.8)	
Divorced or separated	23 (1.4)	4 (1.0)	19 (1.6)		13 (1.6)	10 (1.2)	
Widowed	93 (5.8)	18 (4.5)	75 (6.2)		52 (6.5)	41 (5.0)	
High school education	239 (14.8)	70 (17.5)	169 (14.0)	0.08	118 (14.8)	121 (14.8)	0.16
Health insurance				0.21			0.38
Public health service	56 (3.5)	19 (4.8)	37 (3.1)		30 (3.8)	26 (3.2)	
Medical insurance for urban worker or resident	1014 (62.9)	248 (62.0)	766 (63.3)		512 (64.4)	502 (61.5)	
Rural cooperative medical service	461 (28.6)	106 (26.5)	355 (29.3)		218 (27.4)	243 (29.8)	
Other	62 (3.8)	21 (5.3)	41 (3.4)		26 (3.3)	36 (4.4)	
No insurance	16 (1.0)	5 (1.3)	11 (0.9)		7 (0.9)	9 (1.1)	
**Cardiovascular risk factors, No. (%)**							
Diabetes mellitus	469 (29.1)	142 (35.5)	327 (27)	0.001	249 (31.3)	220 (27.0)	0.05

© 2018 Lu Y et al. *JAMA Network Open.*

Hypertension	1103 (68.5)	270 (67.5)	833 (68.8)	0.63	534 (67.2)	569 (69.7)	0.27
Dyslipidemia	819 (50.8)	204 (51.0)	615 (50.8)	0.94	389 (48.9)	430 (52.7)	0.13
Current smoker	606 (37.6)	164 (41.0)	442 (36.5)	0.11	309 (38.9)	297 (36.4)	0.31
Body mass index, median[a] (IQR)	25 (23.0-27.1)	25 (23.3-27.3)	25 (22.9-27.1)	0.37	25 (23.1-27.0)	25 (22.9-27.3)	0.96
Body mass index[a]				0.24			0.38
≤28	1116 (69.3)	265 (66.3)	851 (70.3)		559 (70.3)	557 (68.3)	
>28	241 (15.0)	62 (15.5)	179 (14.8)		109 (13.7)	132 (16.2)	
Waist circumference, median (IQR), cm	90 (83.0-95.5)	90 (85.0-97.0)	90 (82.5-95.0)	0.01	90 (83.0-95.0)	90 (82.0-96.0)	0.44
**Coexisting Conditions, No. (%)**							
Acute heart failure	15 (0.9)	1 (0.3)	14 (1.2)	0.10	8 (1.0)	7 (0.9)	0.76
Acute Stroke	35 (2.2)	9 (2.3)	26 (2.1)	0.90	16 (2.0)	19 (2.3)	0.66
Fluid retention (lower extremity edema)	84 (5.2)	17 (4.3)	67 (5.5)	0.32	44 (5.5)	40 (4.9)	0.57
Pneumonia	53 (3.3)	11 (2.8)	42 (3.5)	0.49	14 (1.8)	39 (4.8)	<.001
**Medical history, No. (%)**							
Prior myocardial infarction	260 (16.1)	79 (19.8)	181 (14.9)	0.02	137 (17.2)	123 (15.1)	0.24
Prior coronary artery bypass graft	12 (0.7)	1 (0.3)	11 (0.9)	0.18	8 (1.0)	4 (0.5)	0.23
Prior PCI	264 (16.4)	79 (19.8)	185 (15.3)	0.04	137 (17.2)	127 (15.6)	0.37
Prior heart failure	560 (34.8)	124 (31.0)	436 (36.0)	0.07	252 (31.7)	308 (37.7)	0.01
Prior angina pectoris	218 (13.5)	42 (10.5)	176 (14.5)	0.04	114 (14.3)	104 (12.7)	0.35
Prior stroke	227 (14.1)	63 (15.8)	164 (13.5)	0.27	112 (14.1)	115 (14.1)	1.00
**Vital and laboratory results**							

© 2018 Lu Y et al. *JAMA Network Open.*

Systolic blood pressure, median (IQR), mm Hg	130 (120.0-146.0)	132 (120.0-150.0)	130 (120.0-145.0)	0.18	130 (120.0-147.0)	130 (120.0-146.0)	0.61
Heart rate on admission, median (IQR), beat/min	70 (64.0-78.0)	71 (65.0-78.0)	70 (64.0-78.0)	0.63	71 (64.0-78.0)	70 (64.0-78.0)	0.54
**Electrocardiogram findings, No. (%)**							
Rhythm on electrocardiogram							
Atrial fibrillation or flutter	37 (2.3)	9 (2.3)	28 (2.3)	0.94	14 (1.8)	23 (2.8)	0.16
Ventricular tachycardia	3 (0.2)	0	3 (0.2)	0.32	1 (0.1)	2 (0.2)	0.58
Global Registry of Acute Coronary Events risk score, median (IQR)	103 (85.0-121.0)	102 (83.0-120.0)	103 (85.0-121.0)	0.60	100 (84.0-120.0)	105 (86.0-121.0)	0.02
Left bundle branch block	10 (0.6)	2 (0.5)	8 (0.7)	0.72	3 (0.4)	7 (0.9)	0.22
Left ventricular ejection fraction, median (IQR), %	62 (57.0-66.5)	61 (57.0-66.0)	62 (57.0-67.0)	0.49	61 (57.0-66.0)	62 (58.0-67.0)	0.07
Glomerular filtration rate, median (IQR), ml/min/1.73 m^2	79 (69.3-91.2)	80 (67.9-93.1)	79 (69.4-90.8)	0.18	79 (69.5-91.0)	79 (69.0-91.2)	0.99
Number of symptoms, median (IQR), No.	2 (1.0-4.0)	2 (1.0-3.0)	2 (1.0-4.0)	<.001	2 (1.0-3.0)	2 (1.0-4.0)	0.08
**Health status on admission, No. (%)**							
SAQ Physical limitation score, median (IQR)	83 (66.7-100.0)	94 (77.8-100.0)	81 (63.9-95.8)	<.001	86 (68.1-100.0)	83 (63.9-100.0)	0.01
SAQ Physical limitation, No. (%)				<.001			<.001
Minimal (>75)	950 (59)	294 (73.5)	656 (54.2)		496 (62.4)	454 (55.6)	
Mild (>50 to 75)	412 (25.6)	76 (19)	336 (27.7)		207 (26)	205 (25.1)	
Moderate (>25 to 50)	151 (9.4)	10 (2.5)	141 (11.6)		53 (6.7)	98 (12)	
Severe (<=25)	98 (6.1)	20 (5)	78 (6.4)		39 (4.9)	59 (7.2)	

SAQ Angina stability score, median (IQR)	25 (0-50.0)	50 (50.0-50.0)	25 (0-50.0)	<.001	25 (0-50.0)	25 (0-50.0)	<.001
SAQ Angina stability, No. (%)				<.001			<.001
Much better (>75)	149 (9.2)	36 (9)	113 (9.3)		67 (8.4)	82 (10)	
Slightly better (>50 to 75)	46 (2.9)	11 (2.8)	35 (2.9)		22 (2.8)	24 (2.9)	
Unchanged (50)	505 (31.3)	272 (68)	233 (19.2)		290 (36.5)	215 (26.3)	
Slightly worse (25 to <50)	408 (25.3)	49 (12.3)	359 (29.6)		202 (25.4)	206 (25.2)	
Much worse (<25)	503 (31.2)	32 (8)	471 (38.9)		214 (26.9)	289 (35.4)	
SAQ Angina Frequency score, median (IQR)	60 (40.0-80.0)	100 (90.0-100.0)	50 (20.0-70.0)	<.001	70 (40.0-90.0)	60 (30.0-80.0)	<.001
SAQ Angina Frequency, No. (%)				<.001			<.001
None (100)	289 (17.9)	289 (72.3)	0 (0)		184 (23.1)	105 (12.9)	
Monthly (>60 to <100)	418 (25.9)	79 (19.8)	339 (28)		217 (27.3)	201 (24.6)	
Weekly (>30 to 60)	514 (31.9)	27 (6.8)	487 (40.2)		217 (27.3)	297 (36.4)	
Daily (<=30)	390 (24.2)	5 (1.3)	385 (31.8)		177 (22.3)	213 (26.1)	
SAQ Quality of Life score, median (IQR)	58 (41.7-75.0)	67 (50.0-83.3)	50 (41.7-66.7)	<.001	67 (58.3-83.3)	42 (33.3-58.3)	<.001
SAQ Quality of Life, No. (%)				<.001			<.001
Excellent (>75)	447 (27.7)	167 (41.8)	280 (23.1)		387 (48.7)	60 (7.4)	
Good (>50 to 75)	649 (40.3)	145 (36.3)	504 (41.6)		324 (40.8)	325 (39.8)	
Fair (>25 to 50)	390 (24.2)	74 (18.5)	316 (26.1)		74 (9.3)	316 (38.7)	
Very poor to poor (<= 25)	125 (7.8)	14 (3.5)	111 (9.2)		10 (1.3)	115 (14.1)	

Abbreviations: SAQ, Seattle Angina Questionnaire; IQR, interquartile range; PCI, percutaneous coronary intervention.

© 2018 Lu Y et al. *JAMA Network Open.*

a: Calculated as weight in kilograms divided by height in meters squared.

© 2018 Lu Y et al. *JAMA Network Open.*

eTable 3. SAQ score with and without adjusting for missing data

Characteristics	Unweighted	Weighted
**Baseline, median (IQR)**		
Physical limitation at baseline	83.3 (66.7-100.0)	83.3 (63.9-100.0)
Angina stability at baseline	25 (0-50.0)	25 (0-50.0)
Angina frequency at baseline	60 (40.0-80.0)	60 (40.0-90.0)
Quality of life at baseline	58.3 (41.7-75.0)	58.3 (41.7-75.0)
**12-Months, median (IQR)**		
Physical limitation at 12-months	100 (80.6-100.0)	97.2 (79.2-100.0)
Angina stability at 12-months	50 (50.0-50.0)	50 (50.0-50.0)
Angina frequency at 12-months	100 (90.0-100.0)	100 (90.0-100.0)
Quality of life at 12-months	66.7 (50.0-87.5)	75 (50.0-91.7)

Abbreviations: SAQ, Seattle Angina Questionnaire; IQR, interquartile range.

© 2018 Lu Y et al. *JAMA Network Open.*

eFigure 1. Flowchart of study population selection

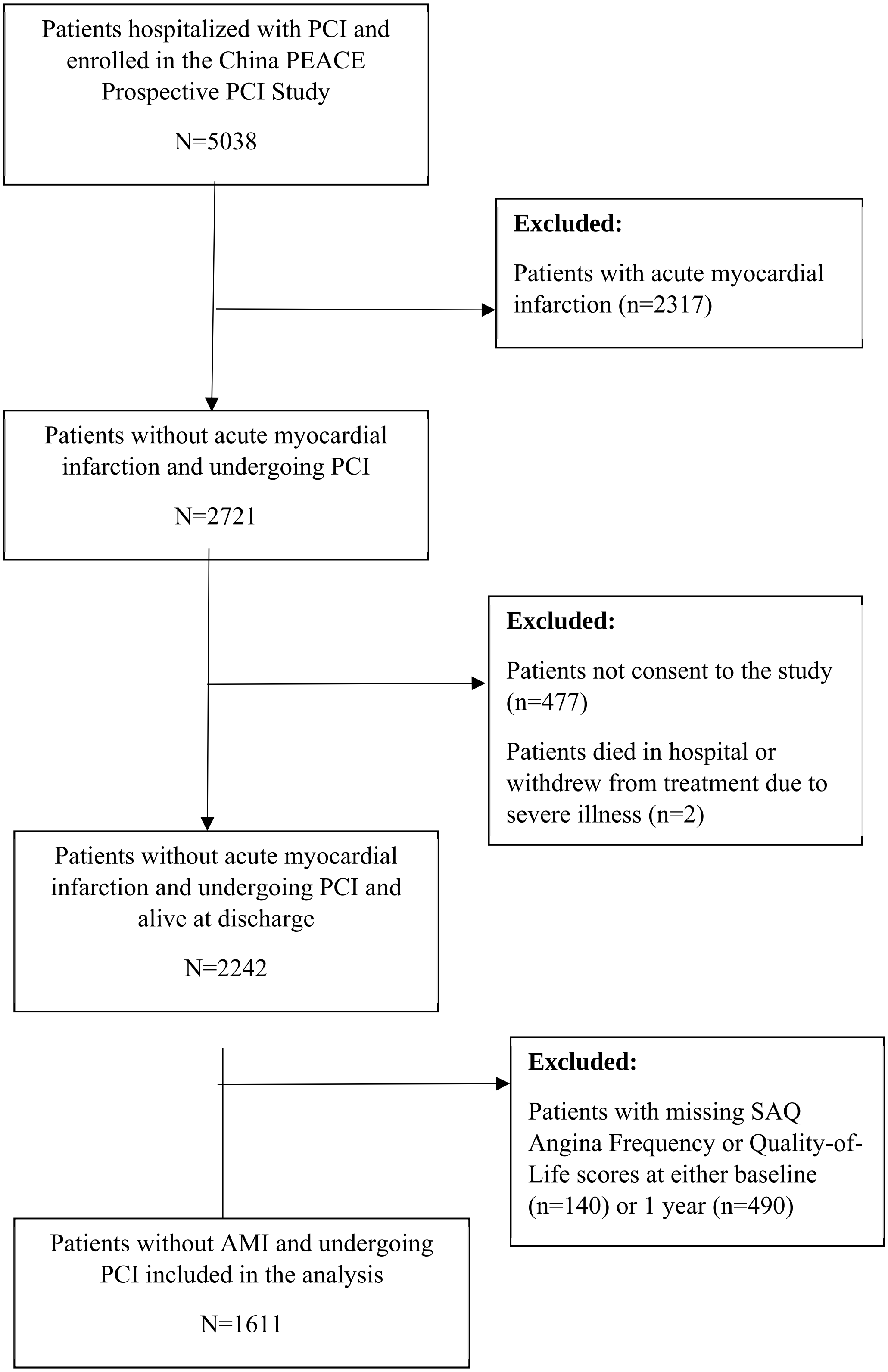

Abbreviation: SAQ, Seattle Angina Questionnaire.

© 2018 Lu Y et al. *JAMA Network Open.*

eFigure 2. Density plot of 1-year change in SAQ Angina Frequency and Quality-of-Life scores

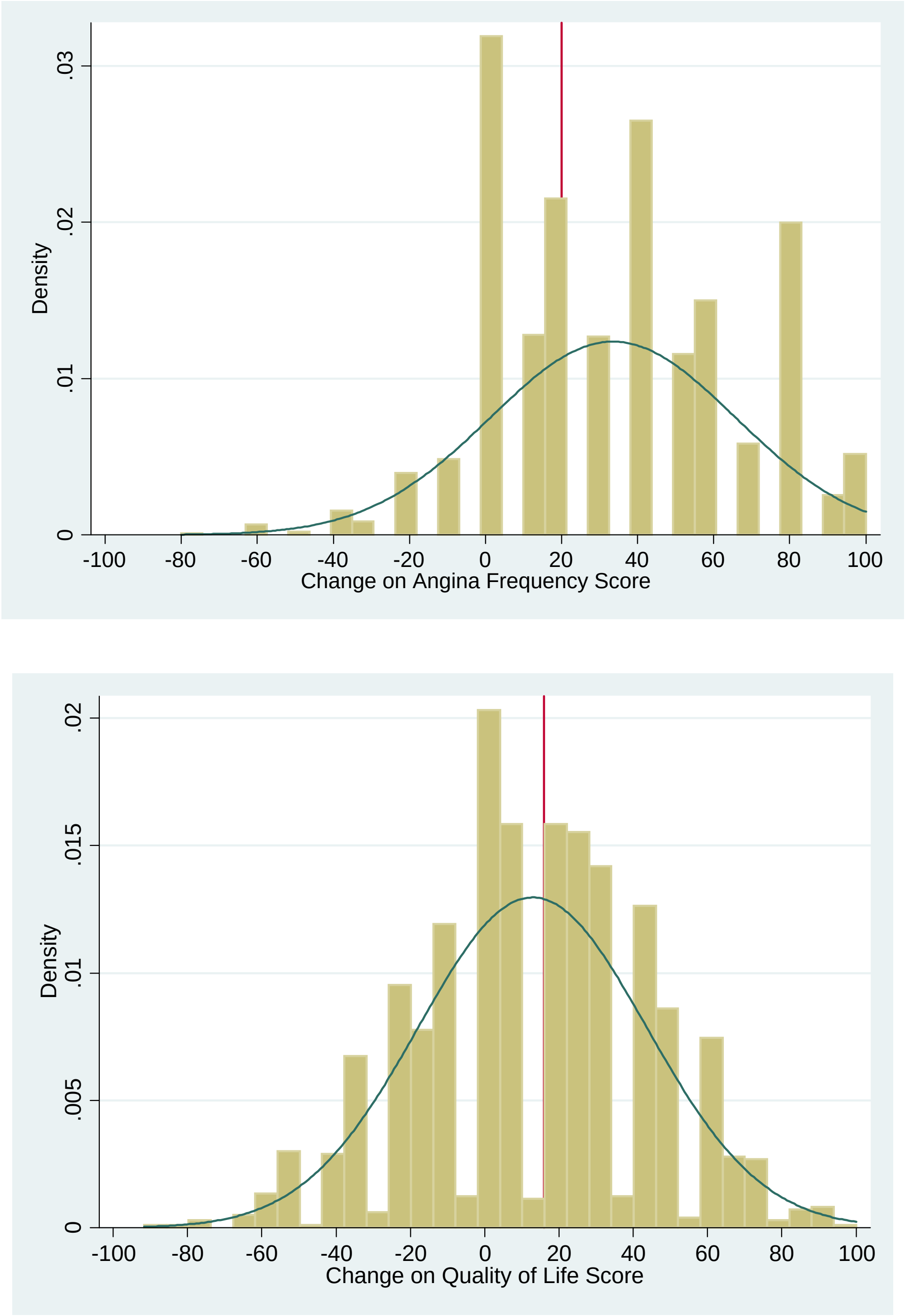

Abbreviation: SAQ, Seattle Angina Questionnaire.

© 2018 Lu Y et al. *JAMA Network Open.*
